# Parental experiences and needs of caring for a child with 22q11.2 deletion syndrome

**DOI:** 10.1186/s13023-023-02980-3

**Published:** 2023-12-04

**Authors:** Dariusz Walkowiak, Jan Domaradzki

**Affiliations:** 1 Department of Organization and Management in Health Care, Poznan University of Medical Sciences, Przybyszewskiego 39, Poznań, 60-356 Poland; 2 Department of Social Sciences and Humanities, Poznan University of Medical Sciences, Poznań, Poland

**Keywords:** Rare Diseases, 22q11.2DS, Caregivers, Children, Parenting, Healthcare, Online survey

## Abstract

**Background:**

For a variety of reasons, raising a child with 22q11.2DS has significant psychosocial and financial repercussions for the family caregivers. Our aim was to identify and explain the expectations and concerns of Polish parents of 22q11.2DS children. An online survey was developed consisting of four sections: demographics, emotions experienced by caregivers while performing their duties, attitudes of the respondents about providing care, and finally different aspects of the caregivers’ life satisfaction. The study was conducted with the support of the Polish 22q11 Association.

**Results:**

Forty-four caregivers of Polish origin completed the survey, all but one of whom were mothers. Thirty-four per cent (n = 15/44) declared full-time employment. According to 73% (n = 32/44) of those surveyed, the child’s disease has not harmed their relationship with the partner. In spite of the fact that the median diagnosis time was 1.9 years (ranging from 0 to 12 years), the caregivers indicated that they had contacted on average 3.9 doctors before obtaining the right diagnosis (range 1–17). The Internet was the main source of information and knowledge about their child’s disease for 93% of respondents (n = 41/44), while for 54% (n = 24/44) it was the association for people with 22q11DS. Only 26% rated as very good or good the support for caregivers offered by the central and local government or its agendas. The physicians’ knowledge about 22q11DS was positively rated by 14% of respondents (n = 6/44). The most frequently chosen source of support for 66% of respondents (n = 29/44) turned out to be their families, and for 34% – a Facebook support group (n = 15/44). Asked how often they rated their quality of life (QoL) highly, none of our respondents chose the option “always”, although 64% (28/44) gave the answer “often”.

**Conclusion:**

Our study is the first one in Poland to develop an online survey specifically for use with caregivers of paediatric patients with 22q11.2DS. Our respondents revealed that caring for 22q11.2 children entails a burden that extends far beyond clinical facets and has a significant impact on every dimension of the caregivers’ lives, including their mental health, everyday activities, families, professional career and social lives. At the same time, caregivers are de facto left alone with the bureaucracy of the healthcare system.

## Introduction

The most frequent microdeletion syndrome, chromosome 22q11.2 deletion syndrome (22q11.2DS)(OMIM 192,430, OMIM 188,400), affects 1 in 2,000–4,000 live births and is a rare multisystem genetic disorder [1–3]. 22q11.2 deletion is the most frequent cause of DiGeorge syndrome, as well as several other clinically described conditions (velocardiofacial syndrome, VCFS conotruncal anomaly face syndrome, Cayler cardiofacial) and a subset of patients with Opitz G/BBB syndrome [1]. It is caused by a small piece of chromosome 22 known as 22q11.2 missing resulting in loss of ~ 50 genes [2]. Phenotypic expression can range from severe, life-threatening condition to simply a few related traits, and it is very changeable. The most typical medical issues in childhood include: congenital heart defects, in particular conotruncal anomalies; palatal abnormalities, most frequently velopharyngeal incompetence (VPI); immunodeficiency; hypocalcaemia due to hypoparathyroidism; genitourinary anomalies; severe feeding/gastrointestinal differences; and subtle dysmorphic facial features. 22q11.2DS is linked to a high risk of neuropsychiatric disorders such as schizophrenia, intellectual disability, attention-deficit hyperactivity disorder (ADHD), autism spectrum disorder (ASD), anxiety and mood disorders, seizures and early-onset Parkinson’s disease [1, 2, 4–9]. In children with the 22q11.2 deletion syndrome, mortality rates are higher and there is considerable multimorbidity [10].

Even though 90% of people with 22q11.2DS have the same deleted region, neuropsychiatric consequences vary greatly between people and across the lifespan [11]. It is yet to be proven that genotype and phenotype have a definite relationship. Genetic background effects, extra-rare pathogenic mutations and potential regulatory roles for certain genes in the 22q11.2 deletion area are some of the phenomena that might account for this variability. Genetic analysis must be used to make the final determination [11]. While few examples of autosomal dominant inheritance (8–28%) have been reported, 90% of DiGeorge syndrome cases are de novo [12].

Highly variable, both within individuals and during the course of development, is also the neurocognitive profile. Motor delays (sometimes accompanied by hypotonia) and speech/language difficulties are frequently seen starting in infancy. Learning challenges are fairly typical in preschool and elementary school children [2, 13]. The IQ distribution in children and teenagers with 22q11.2DS is very variable and similar to the IQ distribution in the general population, but it is displaced about 2 standard deviations to the left [14]. About one-third of patients with 22q11.2DS have mild to moderate intellectual disability, while the rest have IQs that lie in the borderline range (70–84) [2]. Decline in verbal IQ over time is common [15, 16]. Most 22q11.2DS children had impaired language skills [17]. Except for secondary insults such as hypoxia ischemic events during heart repair, or congenital brain malformations like polymicrogyria, more severe levels of intellectual disability are uncommon in children and adolescents but more common in adults.

As a result, patients with this condition may experience severe difficulties adjusting to normal life. This feature is highlighted by the degree of dependence required to carry out daily tasks like talking, owing to palate abnormalities, and walking or participating in sports without restriction – due to cardiac symptoms [18]. Other problems that frequently have a detrimental effect on the lives of these people include difficulties adjusting to the surroundings and difficulties integrating into school or the workplace in adult life [11]. Generalists and specialists in a variety of professions are needed to provide paediatric treatment for children with 22q11.2DS in order to understand the overall linked consequences of the accompanying medical and developmental traits and their impact on well-being and quality of life. It is crucial to have a basic understanding of varying expressivity, the severity of traits, changes with time, and the importance of family-centered treatment [1].

For the above reasons, raising a child with 22q11.2DS has significant psychosocial and financial repercussions for the family caregivers. These issues can, in turn, impair the caregivers’ capacity to look after the affected children. However, whereas Polish researchers tend to concentrate on the clinical aspect of 22q11.2DS [19, 20], with patients as the centre of professional attention, 22q11.2DS caregivers are frequently ignored. The objective of the present study is to identify and explain the expectations and concerns of Polish parents of 22q11.2DS children.

## Materials and methods

The data was gathered via an anonymous, self-administered, online questionnaire on the psychosocial effects of 22q11.2DS on family caregivers between October 28, 2022 and November 28, 2022 among caregivers of children with the disorder. The recruitment effort focused on family caregivers who looked after children with 22q11.2DS (1–18 years old). The study was conducted with the support of the Polish 22q11 Association (in Polish: Stowarzyszenie 22q11 Polska), a non-profit organization founded by parents of children with 22q11 syndrome in 2017 to support people with this condition and their families.

In the study we followed the principles outlined in the Declaration of Helsinki. The Poznan University of Medical Sciences Bioethics Committee examined and authorized the study (KB − 833/22), and each survey respondent gave their informed consent.

No tool has yet been devised to measure the burden carried by the caregivers of children with 22q11.2DS, therefore this survey was conducted using a novel questionnaire that was built on the basis of our study objectives and of the analysis of themes recurrent in literature on the topic. The questionnaire was created according to the guidelines of the European Statistical System [21]. A sociologist, a public health expert and a paediatrician were among the research professionals who first went over the list of concerns voiced by those raising a child with 22q11.2DS. A standard questionnaire was afterwards created. Six items were reformulated as a result of the pilot testing of the questionnaire with five parents. Based on the results, the survey was modified.

There were four sections in the survey. The first set of questions focused on the demographics of the caregivers. The second component of the survey included questions about the emotions experienced by caregivers while performing their duties. The third section of the survey centred on the attitudes of the respondents about providing care, as well as on their perception of the responsibility and burden involved. The caregivers’ life satisfaction was the focus of the fourth section.

The participants were informed about the purpose of the study, its voluntary, anonymous nature, and its confidentiality. They were also given the option to end the interview at any time and to withhold information about their personal circumstances due to the sensitive nature of the topic, which could have resulted in moral harm to study participants. A survey was placed on an internet platform and electronically distributed once for each caregiver after informed consent was received from everyone who volunteered and was included in the study. In addition, non-respondents received two follow-up communications. The survey was devised so as to be possible to complete in about 15–20 min.

The questionnaire data was validated for accuracy, completeness, and consistency before being exported into the statistical program JASP (Version 0.17.1.0). Descriptive statistics were used to present the findings.

## Results

The questionnaire was completed by 44 caregivers in total (mean age: 37.3; range: 25–53), 43 of whom were women (97.7%). All participants were of Polish descent (see Table 1). Only one caregiver (2.3%) looked after more than one child, whereas the majority (97.7%) were caring for just one 22q11.2DS child. Boys outnumbered girls in the 22q11.2DS population (53.3% vs. 46.7%; mean age: 7.3; range: 1–17). The majority of parents indicated that their children had severe (18.7%) or very severe (68%) health issues. 80% of parents of 22q11.2DS children reported not using any kind of extracurricular assistance, whereas 89.3% said they received a care allowance. Exactly half of our group did not work due to caring for a sick child, and additionally two respondents were retired, so they were not active professionally either. As many as 41 (93.2%) people in our group were members of a support group for peeple caring over a 22q11DS child.

**Table 1:** Socio-demographic characteristics of 22q11DS caregivers

Characteristics	N (%)
*Caregiver’s sex*	
female	43(97.7)
male	1(2.3)
*Caregiver’s age*	
Range	25–53
M(SD)	37.3(6.4)
*How many of your children experience 22q11DS?*	
1	43(97.7)
2 or more	1(2.3)
*Child’s sex*	
female	21(46.7)
male	24(53.3)
*Child’s age M(SD)*	
Range	1–17
M(SD)	7.3(4.8)
*How would you rate your child’s health problems*	
very severe	21(47.7)
severe	9(20.5)
moderate	10(22.7)
mild	4(9.1)
none	0(0)
*How many hours per week do you use extracurricular help for your 22q11DS child?*	
1–6	7(15.9)
7–15	6(13.7)
<16	2(4.5)
I do not use any extra help	29(65.9)
*Do you receive care allowance?*	
yes	32(72.7)
no	12(27.3)
*Professional activity*	
unemployed	0(0)
unemployed due to childcare	22(50)
pension	2(4.5)
employed part-time	5(11.4)
employed full-time	15(34.1)
*Has your child’s disease affected a disorganisation of the relationship with the second parent?*	
the child’s disease has not harmed the relationship	32(72.7)
the child’s disease has harmed the relationship with the partner but has not lead to its breakup	9(20.5)
the child’s disease has strengthened the relationship	0(0)
the relationship broke up after the diagnosis was made	0(0)
the relationship broke up as a result of challenges related to caring for *22q11DS* child	1(2.3)
other	2(4.5)
*Are you a member of a support group for persons caring over 22q11DS person?*	
yes	41(93.2)
no	3(6.8)

In spite of the fact that the median diagnosis time was 1.9 years (ranging from 0 to 12 years), caregivers indicated that they had contacted on average 3.9 doctors before getting the right diagnosis (range 1–17, see Table 2). For the vast majority (93.2%) of our respondents, Internet is a source of information and knowledge about their child’s disease, while about half of the caregivers (54.5%) mentioned associations for people with 22q11DS. Specialists were indicated by only 15 respondents (34.1%), and family physicians – by merely 7 (15.9%).

**Table 2:** Diagnostic odyssey in 22q11DS

	N (%)
*How long did it take to obtain a diagnosis? (in years)*	
Range	0–12
M(SD)	1.9(2.8)
*How many physicians did you consult before receiving diagnosis?*	
Range	1–17
M(SD)	3.9(3.9)
*Source of information on 22q11DS*	
Internet	41(93.2)
medical specialist	15(34.1)
family doctor	7(15.9)
local support group	20(45.4)
genetic clinic	12(27.3)
scientific publications	13(29.5)
association/foundation for people with 22q11DS	24(54.5)
other (Facebook, friends with 22q11DS children)	5(11.4)

As many as 27 people from our study group (61.3%) rated the support for caregivers from the government and social institutions rather badly or very badly (see Table 3), whereas 22 respondents (50%) rated the quality of medical care for a 22q11DS child as rather or very good. Access to information on the disease was rated negatively by 30 respondents (68.2%); at the same time, 36 respondents (81.8%) negatively rated physicians’ knowledge about 22q11DS. In the case of the question regarding the general assessment of contacts with the healthcare system, those who had no opinion on this subject were the most numerous (21 respondents – 47.7%).

**Table 3:** 22q11DS caregivers perception of healthcare services

How do you rate the healthcare services for *22q11DS* children	Very bad	Rather bad	I do not know	Rather good	Very good
Support for caregivers from government and social institutions	10(22.7)	17(38.6)	5(11.4)	10(22.7)	2(4.5)
Quality of medical care for your *22q11DS* child	1(2.3)	14(31.8)	7(15.9)	20(45.4)	2(4.5)
Access to specialists (neurologist, geneticist, genetic clinic, psychologist	9(20.5)	20(45.4)	2(4.5)	12(27.3)	1(2.3)
Access to medications for *22q11DS* children	19(43.2)	15(34.1)	7(15.9)	2(4.5)	1(2.3)
Access to and financial help with rehabilitation for *22q11DS* children	2(4.5)	7(15.9)	11(25)	14(31.8)	10(22.7)
Access to information on *22q11DS*	14(31.8)	16(36.4)	3(6.8)	9(20.5)	2(4.5)
Support for *22q11DS* children and caregivers from healthcare professionals	9(20.5)	16(36.4)	8(18.2)	10(22.7)	1(2.3)
Physicians’ knowledge about *22q11DS*	17(38.6)	19(43.2)	2(4.5)	6(13.6)	0(0)
Physicians’ practical information about *22q11DS*(how to provide care for your *22q11DS* child; how to perform various tasks)	14(31.8)	14(31.8)	11(25)	5(11.4)	0(0)
Physician’s/neurologist’s/geneticist’s communication skills	5(11.4)	13(29.5)	4(9.1)	21(47.7)	1(2.3)
Support caregivers receive from physicians	9(20.5)	13(29.5)	12(27.3)	9(20.5)	1(2.3)
Physicians’ empathy	5(11.4)	12(27.3)	7(15.9)	18(40.9)	2(4.5)
Drug and healthcare expenses	7(15.9)	5(11.4)	15(34.1)	7(15.9)	10(22.7)
Adaption of home to the child’s needs	21(47.7)	12(27.3)	5(11.4)	2(4.5)	4(9.1)
Lack of access specialised care equipment	16(36.4)	13(29.5)	10(22.7)	3(6.8)	2(4.5)
Drug reimbursement or purchase of drugs	16(36.4)	13(29.5)	9(20.5)	2(4.5)	4(9.1)
Contact with genetic clinic	3(6.8)	6(13.6)	10(22.7)	24(54.5)	1(2.3)
Contact with psychological clinic	3(6.8)	8(18.2)	16(36.4)	14(31.8)	3(6.8)
Contacts with the healthcare system	1(2.3)	3(6.8)	21(47.7)	10(22.7)	9(20.5)

A significant group of those surveyed (59.1%) noticed the impact of their child’s disease on relations with their family (see Table 4), and 21 of them (47.7%) was aware of the influence on the relationships with healthy children. There were 36 respondents in the surveyed group (81.8%) who worried over the progress of their child’s disease and the possible development of new symptoms. When asked about their happiness, as many as 30 respondents (68.1%) answered positively. None of our respondents assessed their quality of life as very good, although as many as 28 of them (63.6%) considered it to be rather good.

**Table 4:** Feelings toward caregiving

	Never	Rarely	Sometimes	Often	Always
How did your child’s disease affected your relationship with your family?	2(4.5)	4(9.1)	11(25)	20(45.5)	6(13.6)
How did your child’s disease affected your relationship with a healthy child/children?	2(4.5)	13(29.5)	5(11.4)	11(25)	10(22.7)
Do you worry that your other (or future) children can also develop 22q11DS?	16(36.4)	7(15.9)	6(13.6)	5(11.4)	10(22.7)
Do you worry over family finances?	1(2.3)	8(18.2)	10(22.7)	12(27.3)	13(29.5)
Do you feel uncomfortable when other people are in the presence of your 22q11DS child?	16(36.4)	10(22.7)	10(22.7)	8(18.2)	0(0)
Do you worry over the progress of your child’s disease and the development of new symptoms?	0(0)	2(4.5)	6(13.6)	17(38.6)	19(43.2)
Are you bothered by the thoughts on your child’s death?	6(13.6)	13(29.5)	11(25)	9(20.5)	5(11.4)
Have you ever faced stigmatisation resulting from your child’s disease?	20(45.4)	12(27.3)	9(20.5)	3(6.8)	0(0)
Have you ever experienced discrimination resulting from your child’s disease?	19(43.2)	13(29.5)	8(18.2)	4(9.1)	0(0)
Is child’s disease a source of social exclusion?	22(50)	7(15.9)	8(18.2)	7(15.9)	0(0)
Is caregiving a source of satisfaction?	7(15.9)	8(18.2)	11(25)	12(27.3)	6(13.6)
Are you happy?	3(6.8)	5(11.4)	6(13.6)	24(54.5)	6(13.6)
How often do you rate your quality of life (QoL) highly?	1(2.3)	9(20.5)	6(13.6)	28(63.6)	0(0)

In the surveyed group, 23 people (52.3%) agreed with the statement that they can often or always count on emotional support from their family (see Table 5). Among the sources of support in general, the family turned out to be the most frequently indicated (29 respondents – 65.9%), followed by Internet/Facebook support groups (selected by 15 respondents – 34.1%). At the same time, only 8 respondents (18.1%) reported being (always or often) able to count on the support of their physician, and 5 (11.4%) – on the support of other medical personnel.

**Table 5:** Social support for 22q11DS caregivers

	Never	Rarely	Sometimes	Often	Always
*While caring for my* 22q11DS *child*					
I can count on emotional support from my family	2(4.5)	6(13.6)	13(29.6)	10(22.7)	13(29.6)
I can count on emotional support from relatives/friends	9(20.5)	11(25)	14(31.8)	8(18.2)	2(4.5)
I can count on practical help from my relatives/friends (i.e. shopping, cleaning)	12(27.3)	6(13.6)	9(20.5)	10(22.7)	7(15.9)
I have a feeling that my family isolates itself from us	18(40.9)	9(20.5)	11(25)	4(9.1)	2(4.5)
I experience conflicts in my family	18(40.9)	8(18.2)	11(25)	6(13.6)	1(2.3)
*Source of support for* 22q11DS *children and caregivers*					
family	2(4.5)	5(11.4)	8(18.2)	9(20.5)	20(45.4)
relatives/friends	12(27.3)	14(31.8)	8(18.2)	9(20.5)	1(2.3)
associations/foundations for people with 22q11DS	16(36.4)	9(20.5)	10(22.7)	6(13.6)	3(6.8)
psychologist	25(56.8)	8(18.2)	4(9.1)	4(9.1)	3(6.8)
local support group	30(68.2)	6(13.6)	4(9.1)	3(6.8)	1(2.3)
Internet/Facebook support group	5(11.4)	4(9.1)	20(45.4)	11(25)	4(9.1)
neighbours	33(75)	4(9.1)	6(13.6)	1(2.3)	0(0)
physician	10(22.7)	9(20.5)	17(38.6)	6(13.6)	2(4.5)
other medical personnel	23(52.3)	12(27.3)	4(9.1)	4(9.1)	1(2.3)
clergyman	31(70.5)	6(13.6)	3(6.8)	4(9.1)	0(0)
other person	25(56.8)	7(15.9)	7(15.9)	5(11.4)	0(0)
religion/spirituality	20(45.4)	5(11.4)	8(18.2)	8(18.2)	3(6.8)
leisure activity/hobby	9(20.5)	12(27.3)	18(40.9)	2(4.5)	3(6.8)

## Discussion

Rare diseases (RDs) usually impact a child’s emotional and social well-being, and sometimes also their physical growth. In addition to coping with day-to-day issues, parents of children with genetic disorders are concerned about their children’s current functioning and the ability to lead fulfilling adult lives in the future [22–24]. They also have to deal with all the shortcomings of the healthcare system [25]. Given that 22q11DS is one of the more prevalent RDs, our study is relevant to quite a large group of people. If we take into account that some of the analysed issues are universal and also have a bearing on the caregivers of children with other RDs, our findings may prove important for a relatively large group.

Caregivers of 22q11DS patients have to deal with many problems, only some of which are objective in nature. Unfortunately, there are also issues that arise from the way healthcare professionals (HCPs) are educated, or the way the healthcare system is organised, including the availability of various medical procedures or payment for them. For example, only a quarter of our respondents felt that access to information about their child’s disease could be considered satisfactory. This discovery is rather unexpected, particularly considering that all our survey participants identified various online sources as their primary means of acquiring information about 22q11DS. The trend of 22q11DS caregivers seeking information about their children’s condition on the Internet is well-documented in the existing literature [26–28]. However, it is somewhat astonishing that these caregivers find the information they discover to be inadequate. This could potentially be attributed to the scattered nature of information related to DS. However, this may also be related to the lack of exhaustive compendiums of knowledge about the disease. Our findings support earlier findings by Rizzo et al. [27] that the Internet, social media and support groups all play important roles in the development of the knowledge, even if today it seems insufficient. Similarly, in a study by van den Bree et al., the Internet was found to be the most important source of information about manifestations associated with 22q11.2DS [28].

It seems that the length of diagnosis time, as well as the high number of specialists visited by parents with a 22q11DS child before the diagnosis is made, is still a significant and persisting problem [29]. According to our respondents, the average diagnosis time is almost two years. An early diagnosis of the underlying genetic disorder is crucial, and prior research suggests that as children and adolescents with 22q11.2DS get older, there will be an increased need for professional consultation and therapies [30]. Analysing British data, Cohen et al. [31] found that 43% of the patients had been diagnosed as neonates and 12% – before 12 months; in our study these percentages were 23% and 34% respectively. Both studies thus showed that more than 40% of patients with the disease remain undiagnosed after the age of one, though it would seem that the diagnostic process in Poland is somewhat delayed in comparison with the United Kingdom. Obviously, this fact only is sufficient to influence the caregivers’ opinions about the physicians’ knowledge of 22q11DS: among our respondents, as many as 81.8% considered it to be inadequate. The inadequate knowledge can be attributed, at least in part, to the presence of multiple medical conditions within 22q11.2DS, as well as the wide range of biological, psychological, and social circumstances that these children and their families encounter. This coincides with earlier findings of O’Donoghue et al. [32], whose respondents reported insufficient empathy and little awareness of 22q11DS among HCPs; similarly, the parents of 22q11DS patients interviewed by Hallberg et al. [33] often reported a negative approach from HCPs. Admittedly, in our study we only asked about the empathy of physicians, not of all HCPs, but even the opinions we elicited do not indicate too much attention paid by professionals to this issue. There is no set way to explain the 22q11DS to parents; information must be personalized to each individual [33]. Our previous research has shown that HCPs lack knowledge in the field of RDs, an observation confirmed also in the present study with regard to 22q11DS [34, 35]. Thus the question remains how to tailor a message to caregivers if in many cases both knowledge and empathy are lacking. Support is necessary in many issues, for example in communication [36, 37]. Because family problems related to disability intensify with the age of the patient, the growing need for counselling, also in the field of treatment, is indicated [38].

Parental psychological discomfort was found to be linked to a greater overall number of issues in the medical and welfare fields [39]. The concerns of our patients seem to indicate that they also face many challenges, both of an individual and systemic nature. What is worth noting is the fact that patients with 22q11DS are not covered by systemic medical care. Therefore, the treatment of these patients is managed sometimes by university hospitals, on other occasions by the parents themselves. And in the latter case, it is often not covered by health insurance, which is an additional burden on household budgets. Our study did not intend to investigate this issue, but our respondents’ answers allow us to infer that the problem is considerable.

The possibility of mental disease in a child was anxiety-provoking for the parents of children with several medical issues in a previous study [40] in a way that other medical diseases were not. In addition to their fear of the sickness itself, they also worried about the stigma it might entail, worries that were much more intense than those of any stigma they had actually encountered. In our study, the experience of stigma or discrimination was not frequent, but it did occur. Asked whether they feel uncomfortable when other people are in the presence of their 22q11DS child, none of our chose the option “always”, though 18.2% of them reported “often” feeling discomfort. Carrion et al. [41] showed that following their child’s diagnosis with 22q11DS, parents were initially under-informed about the psychiatric risks related to the disorder and of the methods that may be utilized to manage and/or safeguard their child’s mental health. They described how a number of obstacles, such as a lack of mental health assistance, prevented them from increasing their understanding of the psychiatric risk and management options. We must be aware that, as a consequence, this may have an impact on the caregivers’ own psyche, in particular due to the lack of appropriate support from specialists. All the more so that, as one of the mothers wrote to us, the caregivers with children suffering from 22q11DS are not covered by any systemic psychological support: if it is necessary, it must be financed from their own resources.

The disease also shapes families in its own way. This applies to relationships with spouse/partner, healthy children and members of the extended family. In a fairly obvious way, we are talking about the stress of parents associated with the child’s disorder in the first place [30, 39, 42–44]. In the study by Hallberg et al., many participants, particularly the mothers, had already been treated for or had been diagnosed with stress-related disease, including depression or unexplained physical pain [33]. The caregivers were sure that their hard daily life with the child was to blame for their stress-related disorders and that they had expended too much energy in their struggle. Despite the fact that the majority of the parents had previously held full-time jobs, taking care of the child seemed to need more energy than the parents possessed. There was also the issue of caring for healthy siblings [33]. Siblings of a child with 22q11.2DS have lately reported experiencing feelings of guilt and humiliation about the condition [45]. The need for adequate support for the siblings of patients has also been indicated [46].

Briegel et al. [44] found hat most primary caregivers appear to have adequate coping mechanisms – including partnership support – which help them preserve standard levels of life satisfaction. Our study supports these findings: in our survey, the question whether they were happy (always and often) was answered in the affirmative by 68.1% of respondents. Taking into account the child’s disease and the number of adversities they have to face, this is indeed an unexpectedly good result. Our study seems to indicate that this coping mechanism is based on the support of the closest family. We appreciate the role of informal support groups, such as Facebook groups, although due to the way our respondents were recruited (which was exactly via Facebook groups), there may be doubt whether this conclusion can be generalised to the entire population of patients and their families. For our research group, the support of the Facebook group is the second most important, after that of the close family.

### Limitations

The limitations of this research should be taken into account when interpreting the results. Firstly, since there is no register of patients with RDs in Poland, it is not known how many patients suffer from 22q11.2DS. Secondly, we reached patients’ caregivers mainly using Facebook of the Polish 22q11 Association. Therefore, the survey’s findings cannot be applied to the entire group of caregivers in Poland and instead only reflect the views of those 22q11.2DS caregivers who decided to take part in the study. Thirdly, there is a chance that some phrases or questions in this study will be misunderstood because the survey is caregiver-reported. Fourthly, a small number of questions regarding parents’ perceptions of the clinical image of their child’s disease were asked, despite the study’s primary focus being on caregivers’ perceptions of the difficulty associated with caregiving.

## Conclusions

Unfulfilled needs exist for parents who are raising a kid suffering from an RD, and these needs have many distinct and complicated causes. 22q11.2 deletion syndrome is one of the more common RDs that necessitate quite complex healthcare. In the case of Poland, an additional factor is the lack of comprehensive solutions in this area. Our goal was to identify what kind of support would be the most appropriate for the caregivers of children 22q11.2 deletion syndrome. It seems that in the case of this disease, as well as of many other RDs, the issues related to the problems and needs of caregivers of the affected children remain somewhat outside the mainstream of research.

Our respondents revealed that caring for 22q11.2 children has an impact that extends far beyond clinical facets and has a significant impact on every dimension of caregivers’ lives, including their mental health, everyday activities, families, professional career and social lives. At the same time, carers are de facto left alone with the entire healthcare system. Diagnosing the disease takes too long, and in some cases numerous visits to the physicians are necessary. A considerable proportion of the diagnostic process and therapy is financed from the caregivers’ own resources, outside the healthcare system. Most of our caregivers gave up their professional careers and focused on caring for the child. In this situation, private healthcare puts a strain on the family budget. Systemic changes in the care of patients with 22q11.2 are necessary.

Our respondents do not positively assess the knowledge of doctors about their child’s disease, and they are similarly critical of their empathy. The available sources of knowledge about the disease have also been assessed negatively. Also in this respect systemic changes seem necessary.

Finally, awareness must also be raised within the healthcare system that, in addition to the patients themselves, their caregivers need support as well.

## Data Availability

The datasets generated during the study are available from the corresponding author on reasonable request. We are unable to publicly share our data in order to safeguard the privacy of our study participants, as required by EU General Data Protection Regulation (GDPR).

## Abbreviations

22q11.2DS: 22q11.2 deletion syndrome
ADHD: Attention-deficit hyperactivity disorder
ASD: Autism spectrum disorder
RDs: Rare diseases
VPI: Velopharyngeal incompetence
